# Moving Toward Assured Access to Treatment in Microbicide Trials

**DOI:** 10.1371/journal.pmed.0030153

**Published:** 2006-07-11

**Authors:** Anna Forbes

## Abstract

Anna Forbes discusses the Global Campaign for Microbicides' consensus statement on access to treatment and standards of care in microbicide trials.

Public concern about ethics in HIV-prevention trials intensified in the last 18 months, reaching a flashpoint that resulted in the halting of two clinical trials to test the efficacy of tenofovir as a possible method of pre-exposure prophylaxis (PREP) against HIV infection. A reverse transcriptase inhibitor, tenofovir is viewed as a promising candidate for PREP because it is already being widely used as a component in combination-therapy regimens to suppress viral replication, it has a once-daily dosing schedule, and clinical trials have shown a low level of side effects [ 1].

Six randomized, placebo-controlled trials to test tenofovir among high-risk populations have been planned to date. Two of these were halted by government order of the host country: one in Cambodia in August 2004 and one in Cameroon in February 2005. In March 2005, the PREP Nigerian trial was also stopped; but this occurred because, according to the trial sponsor, the local clinical trial site was unable to achieve “the necessary scientific standards.”

Intense controversy among trial sponsors, HIV/AIDS activists, and some trial participants gave rise to these politicized, trial-halting decisions. Voices on all sides have expressed strong opinions – with some charging that the rights and interests of the highly vulnerable populations enrolling in the trials were not adequately protected and that the trials were, therefore, unethical.

One key issue in this debate has been the provision of antiretroviral treatment (ART) to those who seroconvert during the trial—generally referred to as “access to care.” Public perception of researchers' responsibility in this area is rooted in the physician's traditional obligation to provide research participants with the best possible care. What this tradition does not address, however, is the challenge of meeting such an obligation in an environment of massive inequalities.

The Joint United Nations Programme on HIV/AIDS (UNAIDS) has held several regional and international deliberations in the last seven years that addressed (among other issues) the question of access to care. In July 2003, they convened “HIV Treatment for Intercurrent Infections,” a consultation in which vaccine and microbicide researchers came together with social scientists, ethicists, community representatives, and donors. Together they affirmed that providing ART conforms to several fundamental ethical principles: *beneficence*, by which researchers are obliged to maximize benefits to participants; *reciprocity*, which suggests that those who contribute important data to the study by becoming infected deserve something in return; and *justice*, which requires that all seroconverting trial participants be treated equally, regardless of location [ 2]. [Fig pmed-0030153-g001]

**Figure pmed-0030153-g001:**
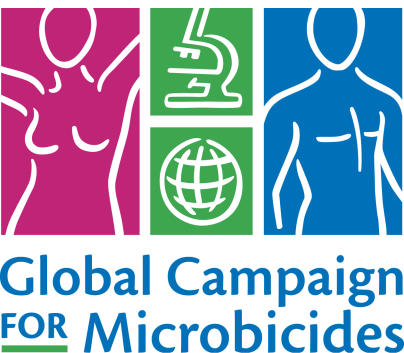
(Image: Global Campaign for Microbicides)

Some bioethicists argue that the provision of ART to those who seroconvert during an HIV-prevention trial is ethically obligatory. Others contend that it does not qualify as a mandatory obligation but that it is, nevertheless, “morally praiseworthy”—something that *should* be done if at all possible. Despite these differing definitions, existing social and political realities have now rendered provision of access to care a critical factor in a trial's ability to go forward.

## Why the Global Campaign for Microbicides Is Involved

The Global Campaign for Microbicides is a broad-based international coalition working to accelerate access to new HIV-prevention options. We mobilize the civil society arm of the microbicide movement by serving as a unifying umbrella under which activists, nongovernmental organizations and other civil society voices work collectively to advance this common goal.

Since its inception in 1998, the Global Campaign has helped lead the search for practical solutions to the ethical quandaries inherent in microbicide trial design and implementation. Faced with the possibility that microbicide trials could be shut down if the issue of access to care was not adequately addressed, we saw the need to articulate a clear position. We developed this position to address the ethical tensions that have surfaced in a way that both responds to community concerns and contributes to resolving the impasses that have resulted in trial closures.

Chief among these is the question of *how* a sponsor can effectively assure access to care in the developing world to someone who may not need it for a decade or more. Care providers, even major international development agencies, may come and go. Trial sponsors also come and go. But providing expanded access to treatment is not a transitory commitment. As one UNAIDS consultant noted, “A lot of these sponsors are ready to make the commitment financially [to fund future access to care to seroconverting trial participants]. But now they're saying, “'Who do I write the check to?'” (E. McGrory, personal communication).

## Drafting a Consensus Statement

The Global Campaign Consensus Statement is informed by structured discussion and debate among Global Campaign partners, allies, staff, and steering committee members during three consultations (a much more detailed report on the results of these deliberations is available in [ 3]). The Statement's consensus points are shown in Box 1, and a longer version (which includes details of the origin of the consensus, a list of organizational endorsers, and a list of the Global Campaign for Microbicides' steering committee members) is provided in Text S1.

Drafted in June 2005, the Statement was first presented at a UNAIDS meeting on “Creating effective partnerships for HIV-prevention trials in developing countries.” Held in Geneva June 20–21, the meeting brought together 70 stakeholders from 20 countries—a diverse range of civil society representatives, activists, researchers, government officials, trial sponsors, and international organizations.

As the report of this meeting notes, “the context of clinical research on HIV has changed. Activism has empowered previously marginalized populations to articulate and demand their rights. Changes in possibilities and expectations around providing treatment for HIV-related disease have also profoundly shifted the context; affected communities expect access to treatment, and are demanding that service providers, community groups, governments and donors respond” [ 4].

The field as a whole now faces the difficult challenge of moving from generalities to specifics regarding how this demand will be met. We drafted the Consensus Statement to start the field-wide process of articulating those specifics.

## The Challenges of Implementation

The pathway for operationalizing access to care is far from clear, but several steps down that path are being taken by various entities.

One approach to ensuring that people who seroconvert during the course of a microbicide trial have access to HIV care is to initiate trials only in areas where HIV treatment roll out is occurring already and/or where other government-sponsored ART programs are already in place. This approach, under consideration by several trial networks, has its limitations. Such programs may be over-subscribed and participants may be placed in a long queue when they need treatment—rendering them unable to access care in a timely fashion despite theoretical accessibility.

Providing expanded access to treatment is not a transitory commitment.

Some advocates have suggested that each research sponsor contribute toward the cost of government-provided ART at a level commensurate to the number of participants who seroconvert during their trial. Such contributions could help relieve some of the pressure on over-burdened treatment programs and help build local health care infrastructure.

Another approach—and one being considered by at least one major microbicide trial network—is that the sponsor may commit to paying for private care for each seroconverting participant as needed until such time as adequate care becomes available to that participant from a public source in her/his country. While this approach sidesteps the precariousness of trying to expedite access to care through nascent government programs, it also introduces two major difficulties: that of creating a mechanism to set aside funding (a trust, insurance scheme, etc.) to cover the cost of care and that of developing durable contracts for the provision of future care if and when it is needed.

The latter option also raises the specter of undue inducement. Undue inducement occurs if the benefits offered to trial participants are great enough to persuade a potential participant to take a risk (by enrolling in the trial) that a reasonable person would not otherwise take. If trial participation enables seroconverting participants to “jump the queue” and access ARTs promptly either through a government program or through private providers, is that benefit alone sufficient to induce HIV-negative individuals in countries with high HIV sero-incidence to enroll in the trial when they might otherwise not do so?

Cathy Slack, an ethicist from the University of Kwazulu-Natal, points out that data on behavior changes during vaccine and microbicide trials show that risk behavior generally does not increase among enrolled trial participants. Indeed, it is more likely to decline as the trial progresses [ 5]. Given this, people choosing to participate in the trial (whether to obtain access to ARTs if they convert or for other reasons) are not, in fact, taking a risk that a reasonable person would avoid. The trial itself does not increase participants' HIV risk, provided that the informed consent process is done properly and the participant is aware of the fact that the candidate microbicide cannot be relied upon for protection.

In developing this Consensus Statement, it was clear that we could not unravel all the complexities associated with negotiating access to care. For this reason, a key consensus point is that

“UNAIDS or another such body should convene a task force of clinicians, people living with HIV/ AIDS, advocates, health economists, legal and insurance experts and entities with relevant experience (such as Pharm Access and Médecins Sans Frontières) to develop and evaluate concrete mechanisms for operationalizing ART access for those who sero-convert during microbicide trials, recognizing that they may not need ART for many years into the future.”

Developing mechanisms through which durable and fair access to ARTs can be assured will require the committed effort of a carefully selected and balanced team of experts. This task force will likely have to reconvene periodically to adjust these mechanisms as established criteria for treatment access change, treatment rollout progresses, and the landscape of health care infrastructure and funding evolves.

But, despite the enormity of the challenge, the endorsers of the Consensus Statement agree that it must be undertaken without delay. Prevention trials must go forward. For that to happen, access to treatment must be assured to those who seroconvert during the trial. Given these imperatives, we have no choice as a field but to commit ourselves to the dauntingly complex challenge of figuring out how it can be done.

## Lessons Learned

The process of developing and promulgating this Consensus Statement reminds us vividly that bioethical decision making is always a balancing act. Research requires the rigorously controlled implementation of scientific protocols. But failing to involve the host communities and other stakeholders in the trial conceptualization and implementation means risking difficulties with participant enrollment and retention—and the possibility of trial discontinuation.

Community members have every right to demand that sustainable improvements in the local standard of care occur as a result of hosting the trial. Researchers, however, face real constraints including the size and budget of the trial and the availability of support from governments, communities, non-governmental organizations, and local providers. Thus, they cannot be expected to correct all the massive inequities in health care access that the community experiences.

Effective and authentic movement toward higher levels of stakeholder involvement requires time—time for expanded feasibility studies, community preparedness work and “joint literacy” training, a process through which researchers are educated about the realities and priorities of their trial host communities while community members and activists are educated about the realities and constraints of doing research. When it comes to HIV prevention, however, the cost of delay is paid in human lives. Where is the appropriate balance between the urgency of developing new HIV-prevention methods and the need to respect and advance the human rights of trial participants and host communities?

A political consensus has emerged around seroconverting trial participants' right to ART access. Given finite resources, how can funding this commitment to the few who seroconvert be balanced against funding the provision of other kinds of care—especially sexual and reproductive health care—to all trial participants (and possibly to the whole of the host community)? Further, how do we balance this political consensus against the sovereign right of host communities and countries to determine the most advantageous benefits package they can negotiate (which may or may not include primary focus on ART provision)?

Knowing that clinical trials can be stopped by broad-based public opposition provides considerable impetus to action. But neither researchers, nor sponsors, nor activists, nor host community members can unilaterally resolve the questions of how to operationalize access to care—much less balance delicate ethical equations such as those mentioned above. Collaboration and transparency, however difficult to achieve, will be required.

Multilateral advocacy work will also be needed to raise the resources and exert the political pressure required to make full access to care a sustainable reality in trial host communities. Governments can and must be involved in decisions regarding where trials can be conducted and kept constantly informed of trial progress. Involving them not only draws their attention to the need to ratchet up the standard of care in those communities, but also helps to build their commitment to, and support for, microbicide research and development.

The activists and trial host communities who put this issue so forcefully on the agenda must now insist that researchers and sponsors join them in advocating to local governments and international funders for their active and escalating investment in this process. Ethicist Solomon Benatar and Kathy Shapiro, a behavioral scientist, have noted that the fact that “the ideal of first world health care cannot be achieved immediately in developing countries should not be a deterrent to efforts to raise existing levels of care” [ 6].

We are walking a difficult line between two imperatives: responding to the urgency of the HIV epidemic and maintaining rigorous ethical standards in HIV-prevention trials. We need to move forward carefully but quickly on that tightrope, recognizing the fact that it is only by truly listening to each other that we can maintain our balance.

## Supporting Information

Text S1 Full Length Version of the Consensus Points on Access to Treatment and Standards of Care in Microbicides Trials (100 KB DOC).

**Box 1. Consensus Points on Access to Treatment and Standards of Care in Microbicides Trials**

1. Clinical trial sponsors and researchers have a responsibility to ensure appropriate care for any negative health consequences that participants experience as a direct result of trial participation.
2. People who seroconvert during the course of a microbicide trial should be assured access to high quality HIV care, including antiretroviral treatment (ART) when it is needed.
  • Trial sponsors and donors should commit to assuring the availability of such care either directly or through explicit and durable partnerships with other care providers. Such agreements should be formalized in consultation with relevant stakeholders and trial communities before a trial starts.
  • There is no consensus among ethicists as to whether the provision of ART to those who seroconvert during a microbicide trial is ethically obligatory, or “morally praiseworthy” but not mandatory. Nonetheless, we call on the wider microbicides community to ensure access to ART based on ethical aspirations and existing social and political realities.
  • UNAIDS or another such body should convene a task force of clinicians, people living with HIV/ AIDS, advocates, health economists, legal and insurance experts and entities with relevant experience (such as Pharm Access and Médecins Sans Frontières) to develop and evaluate concrete mechanisms for operationalizing ART access for those who seroconvert during microbicide trials, recognizing that they may not need ART for many years into the future.
3. Researchers and sponsors, in collaboration with local and national health authorities, should use microbicide trials as an opportunity to strengthen and improve local standards of care. The minimum objective should be to “ratchet up” care in a stepwise, sustainable fashion to reduce global disparities in access to health care.
4. Explicitly defining each site's standard of care must be a mandatory part of trial planning. Negotiations should include agreement with stakeholders, including relevant community and/or civil society groups, on the package of prevention services provided to participants as well as on what other care will be ensured either through direct provision or through effective referral mechanisms.
  • Referral arrangements should be formalized in writing. Researchers and/or trial sponsors should work to ensure that adequate care is actually received through monitoring and support programs for participants (e.g. transportation, accompaniment programs, etc.).
  • Microbicide trials have a special obligation to attend to the sexual and reproductive health needs of participants, including offering direct provision of safe, appropriate contraception for trial participants. Avoidance of unwanted pregnancy will also improve trial power through participant retention.
5. Trial participants should have preferential access to any test product that is shown to be effective. Although ensuring immediate access is complicated by regulatory, manufacturing and licensing issues, researchers and donors should actively seek to accelerate access to product post-trial through implementation of observational/introductory studies and negotiation with host country governments and product sponsors.

**In addition, the Global Campaign for Microbicides commits itself to:**

1. Advocate relentlessly for the right of trial communities to have preferential access to any product proven safe and effective, while being completely candid with communities about the likely time frame of this access.
2. Emphasize in our advocacy the importance of other aspects of standards of care—especially sexual and reproductive health care and the prevention package offered participants—in addition to HIV care and access to ART.
3. Advocate for the right of host communities and countries to have an authentic voice in decisions around trial-related matters, including negotiations of fair benefits.
4. Work to increase research and ethics literacy among advocates and host communities.

## Abbreviations

ART: antiretroviral treatment
UNAIDS: Joint United Nations Programme on HIV/AIDS
