# Supplementary material for: Moving Toward Assured Access to Treatment in Microbicide Trials
Source: PLoS Med. 2006 Jul 11;3(7):e153. doi: 10.1371/journal.pmed.0030153 (PMC1489187; doi:10.1371/journal.pmed.0030153)
Supplement: Supplementary file 1 [file pmed.0030153.sd001.doc]

**Consensus Points on Access to Treatment and Standards of Care in Microbicides Trials**

*On May 30-31^st^ 2005 the Steering Committee of the Global Campaign for Microbicides (GCM) met for two days in Barcelona to discuss issues of access to treatment and standard of care in microbicide trials. The recommendations below represent the points of consensus that emerged from this dialogue. Please see the reverse side for more information on the process of developing this statement.*

1. Clinical trial sponsors and researchers have a responsibility to ensure appropriate care for any negative health consequences that participants experience as a direct result of trial participation.
2. People who seroconvert during the course of a microbicide trial should be assured access to high quality HIV care, including antiretroviral treatment (ART) when it is needed.
   - Trial sponsors and donors should commit to assuring the availability of such care either directly or through explicit and durable partnerships with other care providers. Such agreements should be formalized in consultation with relevant stakeholders and trial communities before a trial starts.
   - There is no consensus among ethicists as to whether the provision of ART to those who seroconvert during a microbicide trial is ethically obligatory, or “morally praiseworthy” but not mandatory. Nonetheless, we call on the wider microbicides community to ensure access to ART based on ethical aspirations and existing social and political realities.
   - UNAIDS or another such body should convene a task force of clinicians, people living with HIV/AIDS, advocates, health economists, legal and insurance experts and entities with relevant experience (such as Pharm Access and Médecins Sans Frontières) to develop and evaluate concrete mechanisms for operationalizing ART access for those who seroconvert during microbicide trials, recognizing that they may not need ART for many years into the future.
3. Researchers and sponsors, in collaboration with local and national health authorities, should use microbicide trials as an opportunity to strengthen and improve local standards of care. The minimum objective should be to “ratchet up” care in a stepwise, sustainable fashion to reduce global disparities in access to health care.
4. Explicitly defining each site’s standard of care must be a mandatory part of trial planning. Negotiations should include agreement with stakeholders, including relevant community and/or civil society groups, on the package of prevention services provided to participants as well as on what other care will be ensured either through direct provision or through effective referral mechanisms.

- Referral arrangements should be formalized in writing. Researchers and/or trial sponsors should work to ensure that adequate care is actually received through monitoring and support programs for participants (e.g. transportation, accompaniment programs, etc.).
  - Microbicide trials have a special obligation to attend to the sexual and reproductive health needs of participants, including offering direct provision of safe, appropriate contraception for trial participants. Avoidance of unwanted pregnancy will also improve trial power through participant retention.

5. Trial participants should have preferential access to any test product that is shown to be effective. Although ensuring immediate access is complicated by regulatory, manufacturing and licensing issues, researchers and donors should actively seek to accelerate access to product post-trial through implementation of observational/introductory studies and negotiation with host country governments and product sponsors.

**In addition, the Global Campaign for Microbicides commits itself to:**

1. Advocate relentlessly for the right of trial communities to have preferential access to any product proven safe and effective, while being completely candid with communities about the likely timeframe of this access.
2. Emphasize in our advocacy the importance of other aspects of standards of care—especially sexual and reproductive health care and the prevention package offered participants­—in addition to HIV care and access to ART.
3. Advocate for the right of host communities and countries to have an authentic voice in decisions around trial related matters, including negotiations of fair benefits.
4. Work to increase research and ethics literacy among advocates and host communities.

*********

**Origins of this consensus**

These conclusions evolved over a three-year period of structured discussion and debate among Global Campaign partners, allies and staff. They draw on three major consultations organized by the GCM, including:

1. A meeting co-organized with International AIDS Vaccine Initiative (IAVI) in 2003, entitled ***Consultation on HIV Treatment in the Context of Prevention Trials***;
2. A global ***Consultation on Ethical Issues in the Clinical Testing of Microbicides***, which brought together 64 people from 12 countries – including advocates, ethicists, clinical investigators, community members, drug regulatory authorities, and past participants in microbicide trials; and
3. A special two-day meeting of the GCM Steering Committee^[1]^ held in Barcelona in May 2005.

The consensus presented here represents the collective points of agreement that emerged from the May GCM Steering Committee. We are currently circulating this document among our partners for formal institutional endorsement. Until that time it should be considered a work in progress. For more information contact: Lori Heise at [lheise@path-dc.org](mailto:lheise@path-dc.org).

**List of Organizational Endorsers**

ActionAIDS

AIDS Committee of North Bay and Area

Aids Fonds

AIDS Foundation of Chicago

AIDS Taskforce of Greater Cleveland

AIDS Treatment Initiatives (ATI)

Aradia Women's Health Center

Association of Nurses in AIDS Care (ANAC)

Atlantic Centre of Excellence for Women's Health, Dalhousie University

California Microbicides Initiative (CaMI)

Canadian AIDS Society

Canadian Federation for Sexual Health *(formerly Planned Parenthood Federation of Canada)*

Canadian HIV/AIDS Legal Network

Canadian Treatment Action Council (CTAC)

CARE Canada

CARE International UK

European AIDS Treatment Group (EATG)

Global Network of People Living with HIV/AIDS North America (GNP+NA)

Grupo de Trabajo sobre Tratamientos del VIH (gTt)

Grupo Português de Activistas sobre Tratamentos de VIH/SIDA (GAT)

HivNorge (tidligere/former Pluss-LMA)

Interact Worldwide

International Planned Parenthood Federation (IPPF)

Journalists Against AIDS, Nigeria
The National AIDS Trust, UK

Naz Foundation International

Nigerian HIV Vaccine and Microbicide Advocacy Group

Northern AIDS Connection Society

PATH

Planned Parenthood of Connecticut

Positive Women's Network

Positively Women

STI AIDS Netherlands

STOP AIDS NOW!

Voices of Positive Women

Women’s Health Advocacy Foundation

1. The Steering Committee of the Global Campaign for Microbicides is comprised of:

   Deborah Arrindel American Social Health AssociationUSManju ChataniAfrican Microbicide Advocacy GroupGhanaPaddy ConnollyCairdeIrelandKim DicksonDFID NigeriaNigeriaChris EliasPATHUSMarc-André LeBlanc Canadian AIDS SocietyCanadaSheena McCormackUK Medical Research Council Clinical Trials UnitUKPromise MthembuInternational Community of Women Living with HIV/AIDSSouth AfricaMargaret MuganwaSociety of Women and AIDS in AfricaUgandaVimla NadkarniTata Institute for Social SciencesIndiaCory Richards The Alan Guttmacher InstituteUSMoniek Van der KroefAIDS Fonds The NetherlandsShira SapersteinMoriah FundUSLaurie Sylla Connecticut AIDS Education and Training Center USJoan Tallada Grupo de Trabajo sobre Tratamientos del VIH SpainGaye Tharawan Women’s health advocateThailandJanneke Van de WijgertInternational Antiviral Therapy Evaluation CentreThe Netherlands
